# Improving the Diagnosis and Follow‐Up of Chronic Myeloid Leukemia Using Conventional and Molecular Techniques

**DOI:** 10.1002/jcla.70001

**Published:** 2025-02-10

**Authors:** Noor Al‐Huda A. Bahar, Mushtak T. S. Al‐Ouqaili, Nabeel M. Talib

**Affiliations:** ^1^ College of Medicine, Department of Microbiology University of Anbar Ramadi Iraq; ^2^ Anbar Health Directorate Anbar Cancer Center Ramadi Iraq

**Keywords:** BCR‐ABL1 fusion gene, CML, FISH, karyotype, molecular cytogenetic response, qRT‐PCR, the predicted‐FISH

## Abstract

**Background:**

The Philadelphia chromosome (Ph) represented a finding of chronic myeloid leukemia (CML) in most cases which formed from t (9; 22) (q34; q11) resulting in the Breakpoint cluster region‐Abelson tyrosine‐protein kinase1 (BCR‐ABL1) fusion gene. Assuming CCE's inaccuracies in diagnosing CML and FISH's limitations with low BCR‐ABL1 percentages, a Predicted‐FISH (Pred‐FISH) was developed. This model predicts treatment response during follow‐up by integrating qRT‐PCR results, White Blood Cell (WBC) counts, and Cytogenetic Response data.

**Methods:**

Quantitative Real‐Time Polymerase Chain Reaction Analysis (qRT‐PCR), fluorescence in situ hybridization (FISH), and Conventional Cytogenetic Examination (CCE or Karyotyping) have been used in the detection and follow‐up of CML patients. The study included 110 individuals, divided into three groups: 31.82% (35 individuals) were newly diagnosed CML patients, another 22.73% (25 individuals) were healthy control samples, and the remaining 45.45% (50 individuals) were previously diagnosed CML patients.

**Results:**

Include BCR‐ABL1 fusion gene levels detected by qRT‐PCR, Ph chromosome presence t (9; 22) (q34; q11) observed by CCE, and WBC counts. The FISH test, used to confirm disease in new patients before treatment, was compared to CCE results due to its insensitivity in certain conditions. Data from CCE, FISH, qRT‐PCR, and WBC for newly diagnosed patients provided a standard for evaluating the Predicted‐FISH.

**Conclusion:**

The FISH technique excels in disease detection with over 98% accuracy and high sensitivity. QRT‐PCR is effective for monitoring CML and BCR‐ABL1 gene levels, indicating MMR and DMR. CCE, while useful for posttreatment monitoring, is less accurate in measuring treatment response over time.

## Graphical abstract

Fusion Metrix for The FISH Model vs CCE for 50 Patients for three Periods.

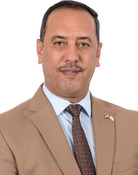

## 1 Introduction

It is well recognized that more than 90% of CML situations are caused by the Philadelphia chromosome (Ph) [1]. Abnormal transmission among t (9;22) (q34; q11) leads to the production of the chimeric fusion gene which represents a marker of CML. Also, this disease is caused by the BCR‐ABL1 gene [2, 3, 4].

Diagnosis of CML disease by techniques like qRT‐PCR, represents the molecular method, while CCE and FISH, represent the cytogenetic methods, which play a crucial role in detecting aberrations and mutations of chromosomes [5, 6]. It is reported that CCE in CML is less specific due to resolution limits, cell heterogeneity, subjective interpretation, and higher detection thresholds [7].

FISH's limited cell sampling (200–500 cells) can miss rare BCR‐ABL‐positive cells, unlike qRT‐PCR, which offers higher sensitivity and quantification of the BCR‐ABL gene [8, 9]. The BCR‐ABL1 gene can be detected via qRT‐PCR even in cases where FISH or CCE analysis fails to detect Ph + due to its lower sensitivity; this finding is in agreement with [10]. Many studies have been done in the modeling aspects like [11], who built a molecular cytogenetic prediction model to forecast cytogenetic abnormalities associated with cancers like multiple myeloma without needing techniques like FISH. Such models rely on gene expression profiles and copy number‐sensitive genes to create a virtual karyotype for predictive purposes.

Therefore, this study has been undertaken to detect Ph by cytogenetic technique and BCR‐ABL1 gene by molecular assay according to the Molecular and Cytogenetic Responses of Table 1 which represent the agreement between the CCE, FISH, and qRT‐PCR data during treatment and follow‐up. It is possible that the FISH equipment does not detect small amounts of chromosome abnormalities during treatment periods contrary to qRT‐PCR. In addition, CCE may give results that do not match qRT‐PCR, Complete Blood Count (CBC) parameters and clinical variables, therefore; Building a predicted model is necessary for accurate assessment that aims to discover a relationship between FISH, qRT‐PCR and CBC factors for enhanced cytogenetic clinical decision‐making.

**TABLE 1:** Molecular and cytogenetic responses during treatment for the BCR‐ABL1 gene and Philadelphia Chromosome [12].

Type of response	Features
Cytogenetic response	
No cytogenetic response (NCyR)	> 95% Ph+
Minor cytogenetic response (MiCyR)	95%–66% Ph+
Partial cytogenetic response (PCyR)	65%–36% Ph+
Major cytogenetic response (MCyR)	1%–35% Ph+
Complete cytogenetic response (CCyR)	< 1% (≤ 1/200) positive interphase nuclei by FISH
Molecular Response	
Complete molecular response (CMR)	No BCR‐ABL1 gene detectable
Major molecular response (MMR)	At least a 3‐log reduction in BCR‐ABL1 or BCR‐ABL1 < 0.1%
Deep molecular response (DMR)	At least a 4‐log reduction in BCR‐ABL1 or BCR‐ABL1 < 0.01%
Major molecular response loss MMR (LoMMR)	Increase in BCR‐ABL1 level above the threshold for MMR

## 2 Patients and Methods

The specimen collection was performed during the period from March‐2023 to Feberuary‐2024 in Anbar Cancer Center and Private clinics in Ramadi City, Anbar Governorate, in addition to Hematology Center, Medical City, and Baghdad.

The study included 110 individuals, divided into three groups: 31.82% (35 individuals) were newly diagnosed CML patients, another 22.73% (25 individuals) were healthy control samples, and the remaining 45.45% (50 individuals) were previously diagnosed CML patients. The overall study population consisted of 60 males (54.55%) and 50 females (45.45%), with approximately a 1.2:1 ratio of male‐to‐female. Furthermore, the mean age of patients was (37.83 ± SD) years.

All patients were monitored at different intervals; The 35 newly diagnosed CML patients were assessed before treatment and with two other subsequent periods of monitoring. These samples were initially detected using four assays (FISH, CCE, qRT‐PCR, and CBC) and subsequently monitored with CCE, qRT‐PCR, and CBC. The 50 previously diagnosed CML patients were monitored during three periods: 3‐, 6‐ and 9‐months posttreatment, using CCE, qRT‐PCR, and CBC assays for assessment. The Table 2 shows the advantages and limitations of each of the techniques used in the study.

**TABLE 2:** The comparison between the study techniques regarding the decision time, Cancer burden, Type of assessment, approximate and limitations for each technique.

Techniques	The time required between sample arrival and result delivery	Cancer burden	Assessments	Approximate cost ($)	Limitations
qRT‐ PCR	5 h	Detection of BCR‐ABL level	Quantitative and qualitative results	200–250	PCR inhibitors and delay in sample processing
FISH	12–15 days	t (9:22) chr. and BCR‐ABL re‐arrangement	Quantitative and qualitative results	400–500	The quality of the sample and processing time
Karyotyping	10–15 days	Microscopic t (9:22) chr.	Qualitative result	250–300	The individual under TKI therapy in addition to sample quality

### 2.1 Ethics Statement

The research applied compliance with the ethical principles regarding the Helsinki Declaration, of 1979, which provides guidelines for conducting medical research relating to members. The study protocol received approval from the Committee for Medical Ethics at the University of Anbar in Ramadi, Iraq, on February 23, 2023 (permission number 140). Oral consent was obtained from all patients participating in the study and the researcher asked the potential participant directly if the individual would like to participate in the study by aspiration of the blood. If the participant agrees to participate, oral consent has been recorded on an audio recorder.

## 3 Cytogenetic and Molecular Techniques

### 3.1 FISH Technique

The procedure of the FISH pretreatment kit achieved according to Vitro Master diagnóstica, Spain, and (cat.no, FISHPTK0056), also BCR‐ABL1 Fusion/Translocation FISH probe, Spain, and (cat.no, 042 M0004). It involves three steps. First, bone marrow slides are prepared, treated with RNase, pepsin, and paraformaldehyde, and dried using ethanol. Second, hybridization: slides receive 30 μL of hybridization solution, are denatured at 70°C, cooled, and hybridized overnight at 37°C. Third, detection: posthybridization, slides are washed, blocked, incubated with Streptavidin‐Cy3, counterstained with DAPI, and mounted. Finally, slides are analyzed using a fluorescence microscope [13, 14, 15].

### 3.2 CCE (Karyotyping)

Kanaan et al. [16] described a lymphocyte culture protocol involving RPMI‐1640 medium supplemented with fetal bovine serum, antibiotics, and phytohemagglutinin. After 72 h of incubation, colchicine was added to arrest cells in metaphase. Hypotonic treatment with KCl, fixation with methanol‐acetic acid, and slide preparation followed, including staining with Giemsa Stain solution. CCE used Meta Class and a fluorescence optical microscope for chromosomal analysis of peripheral blood cultures [17].

### 3.3 QRT‐PCR Technique

The TRUPCR BCR‐ABL1 kit, India and (Cat. No, BCRA‐QT‐M/2024/05) includes RNA extraction from whole blood samples of patients suspected to have CML, mixing blood with Buffer BEL, followed by incubation and centrifugation to isolate the cell pellet. Buffer LR was added, and the lysate was filtered and homogenized. Ethanol was then mixed in, and the sample was transferred into an RNA Binding Spin Column, washed with Buffers WR1 and WR2, and centrifuged to remove contaminants. Finally, RNA was eluted using RNase‐free water. Consequently, the reverse transcription PCR was performed by preparing a reaction mix with RRT components and RNA, followed by thermo cycling through three temperature steps. The resulting cDNA was used for Real‐Time PCR, where the PCR premix and cDNA were combined, mixed, and subjected to thermal cycling as per the set program. The thermal cycler was run at 94°C for 10 min and then cycled at 94°C and 60°C for 45 cycles [18].

Furthermore, The ABL1 and BCR‐ABL1 copy numbers obtained from the test results were used to calculate the Normalized Copy Number (NCN) for samples and the IS Calibrator. The ratio of these values provided the NCN percentage using the formula:

$$\mathrm{NCN}\;\left(\%\right)=\mathrm{BCR}-\text{ABL1}\;\mathrm{CN}/\text{ABL1}\;\mathrm{CN}\times 100\%$$

The cDNA control (High Positive RNA Control) and IS‐MMR Calibrator were used to monitor the reverse transcription and amplification steps of ABL1 and BCR‐ABL1 during transcript quantification. The NCN result was obtained for the IS‐MMR Calibrator (assigned IS Value: 0.243%) with an interval (0.05–0.5). The second calibrator supplied with the kit was assigned a value (Cal NCN% assigned) after calibration against the WHO primary reference standard. The Cal NCN% for the IS‐Calibrator was calculated as described in the formula above. To convert

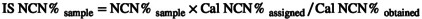




The NCN% result to the international scale (IS NCN %), the formula was applied as follows [19]:

## 4 The Predicted‐FISH Design

### 4.1 Collected Data

Data were collected from 35 patients diagnosed with CML, including the CCE, CBC and qRT‐PCR tests, for predicted model building.

### 4.2 The Predicted‐FISH Process

To design the predicted model, it was fed with various variables data along with FISH data and used Multiple Regression, a statistical method that aims to find relationships for the independent variables among them. These variables are PLT, RBC, HB, WBC, CCE, qRT‐PCR, Blast cell, and Lymphocyte to find variables related to the FISH variable. It was found through numerous attempts that FISH is directly affected by both qRT‐PCR and WBC, unlike the other variables.

### 4.3 Build Correlation

After identifying the relationship of FISH response with the variables qRT‐PCR and WBC, the FISH model was built. The FISH model designed in this study has a limitation as it will be shown later in which WBC must be less than 91000*10^^9^ /L and qRT‐PCR must be larger than 1% to give a good performance and as follows:

(1) $$\begin{array}{c} \text{The Pred‐FISH}=a+(b*\mathrm{qRT}-\mathrm{PCR}\%(+)\;c*\mathrm{WBC}\;(-)\;d*\mathrm{qRT}\\ \;-{\mathrm{PCR}}^{2}(-)\;e*\mathrm{qRT}-\mathrm{PCR}*\mathrm{WBC}) \end{array}$$

Where: FISH: *Fluorescence* In Situ *Hybridization*, %; qRT‐PCR: Quantitative Real‐Time Polymerase Chain Reaction, %; WBC: White Blood Cell, 10^9^ /L; a, b, c, d and e: coefficient of the correlation as in Table 3 below.

**TABLE 3:** Coefficient of the FISH Correlation.

Coefficient	a	b	c	d	e
Value	0.0828	2.617	0.00001	1.47	0.000022

To explain how to apply the coronation equation between the Predicted‐FISH, qRT‐PCR, and WBCs, the following case example has been added: —if the case with CML and the result of BCR‐ABL using qRT‐PCR was 16% and the total numbers of leukocytes were 20,000 cell/mm^3^. Depending on these values and using the following equation: the Predicted‐FISH = a(+) b*qRT‐PCR %(+) c*WBC (−) d*qRT‐PCR^2^(−) e*qRT‐PCR*WBC to detect the Predicted‐FISH values, the predicted‐FISH will be 54%; the patient is classified within the PCyR. While, a qRT‐PCR result of 5% and a total leukocyte count of 8000 cells/mm^3^ yield a Predicted‐FISH result of 20%, indicating that the patient is classified in the major cytogenetic response (MCyR), as described in Table 1.

### 4.4 Calculation Process

**FIGURE 1:**
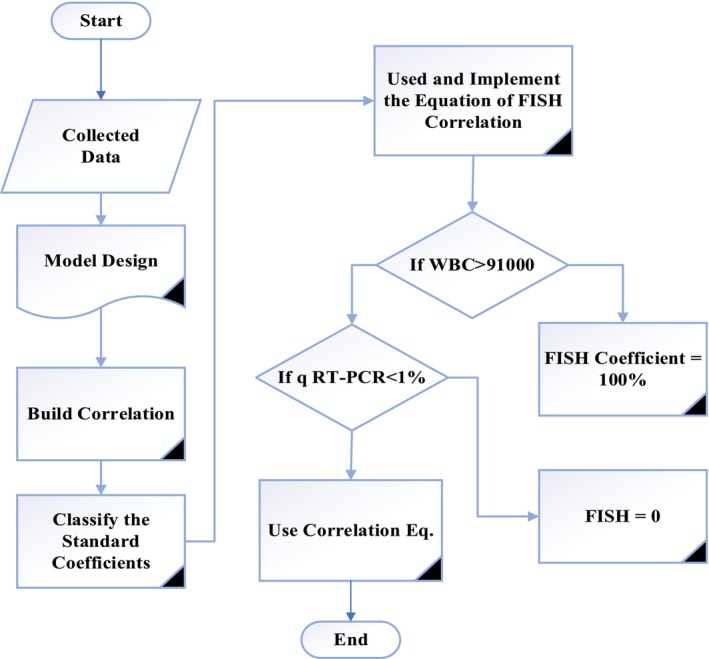
Flow Diagram Represents the Predicted‐FISH.

The input data must be submitted then the role according to correlation limitation for better performance. Where if the WBC is larger than 91,000 10^9^ /L and qRT‐PCR is larger than 1% then the FISH calculated is 100%. Whereas, if qRT‐PCR is less than 1% thus, the FISH is 0%. Otherwise, the correlation is used for FISH calculation (Figure 1).

### 4.5 Model Characterization

**FIGURE 2:**
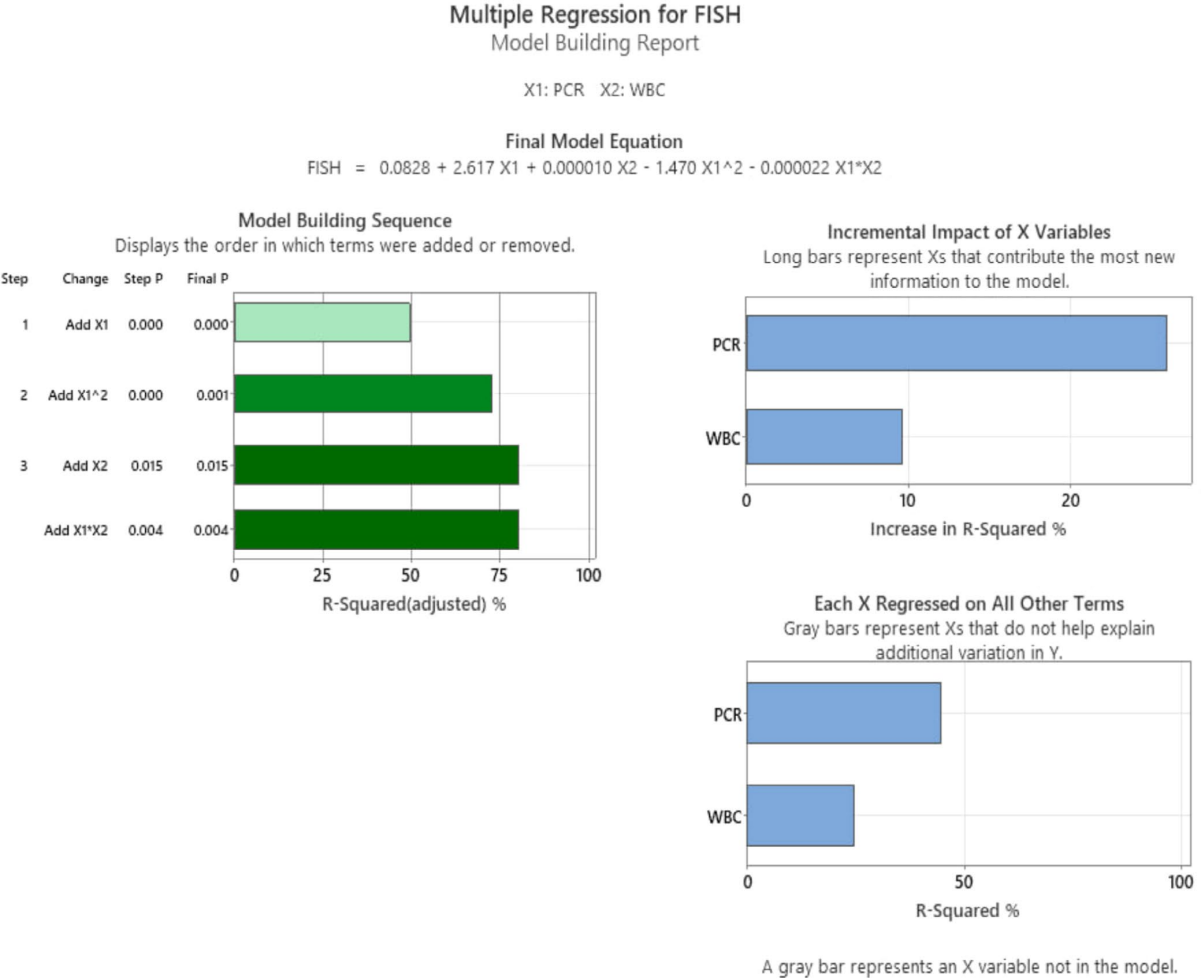
Model Building Report of the Predicted‐FISH Design.

Figure 2 shows the results of a multiple regression analysis that was used to predict FISH values based on qRT‐PCR and WBC values. The analysis found that there is a statistically significant relationship between the X variables (qRT‐PCR and WBC) and the Y variable (FISH) with an *R*‐squared value of (82.70%), which means that (82.7%) of the variation in FISH can be explained by the variation in qRT‐PCR and WBC.

However, it is important to keep in mind that this is just one model, and there may be other factors that could influence FISH results. The Predicted‐FISH analysis could be used to predict the FISH result if the instrument is not available or could be used for comparison side by side with CCE results in a confirmation manner. When the WBC count is < (91,000*10^9^/L), FISH results tend to increase with increasing qRT‐PCR values which is a logical response. On the other hand, when the WBC count is higher than (91,000*10^9^/L), FISH results decrease with increasing qRT‐PCR values. In other words, the constraints of this correlation are valid only for WBC less than (91,000*10^9^/L). Furthermore, there is a significant interaction between qRT‐PCR and WBC on FISH results with a *p*‐value < 0.001. Nevertheless, this analysis suggests that qRT‐PCR is a strong predictor of FISH results, while WBC has a smaller but still significant effect.

### 4.6 Statistical Analysis

The analysis of information from patients with CML was conducted using Microsoft Excel version 16.85, 2024. Additionally, the Predicted‐FISH design was built using Minitab, version: 21.1 (64‐bit), 2021 to enhance the analytical framework. The level of statistical significance was set at < 0.001, adhering to standard practices in research, and statistical decisions were based on the calculated *p*‐values.

## 5 Results

Results in Tables 4, and 5 refer to the detection of the levels of BCR‐ABL1 fusion gene by qRT‐PCR and an observed presence of Ph chromosome t (9;22) (q34; q11) by CCE as well as the WBC for each patient. The difference between Tables 3 and 4 is that the first one includes the laboratory results of the FISH value for new patients before they undergo treatment to confirm the disease, especially since CCE and WBC are not sufficient to confirm the present disease. As for the following table, due to the insensitivity of the FISH test with laboratory instruments as a result of patients' treatment or other reasons, instead the calculated FISH from the Predicted‐FISH outputs was used for comparison with the results of the CCE. Also, the data specific for newly diagnosed patients in Table 4 based practically on the use of CCE, FISH, qRT‐PCR and WBC were used as a golden standard base for the Predicted‐FISH to be more informative.

**TABLE 4:** Detection and monitoring CML newly diagnosed patients with molecular, cytogenetic and total leucocyte count results.

Sample NO.	FISH	CCE Monitoring (Karyotype)			qRT‐PCR Monitoring			WBC (10^9^\L)		
	0 month	0 month	> 3 months	> 6 months	0 month	> 3 months	> 6 months	0 month	> 3 months	> 6 months
1	90%	n/Ph+	n	n/Ph+	49.09%	15.70%	50%	15,000	13,000	17,200
2	99%	n/Ph+	Ph+/n	n	12%	72%	8.76%	20,000	68,000	12,000
3	98%	Ph+	Ph+/n	n	50%	20%	49.65%	54,000	11,000	15,000
4	89%	Ph+/n	Ph+	n	12.92%	5.87%	0%	83,000	6000	9000
5	99%	Ph+	Ph+/n	n/Ph+	22.20%	9.88%	3.00%	16,000	9000	10,000
6	85%	Ph+	Ph+/n	n	51%	23.30%	0.50%	22,000	12,000	11,000
7	80%	n	n	n	62%	20%	0.00%	200,000	30,000	12,000
8	85%	Ph+	n	n	100%	23.30%	0.00%	33,000	15,000	9000
9	99%	n/Ph+	n	n	13.40%	5.50%	0.00%	13,400	8000	8000
10	90%	n/Ph+	n	n	10.10%	0.00%	0.00%	88,000	10,000	11,300
11	99%	n/Ph+	n	n	50%	15.45%	0.70%	91,000	6000	6500
12	90%	Ph+/n	n/Ph+	n	33%	12.11%	0.00%	17,000	8000	11,000
13	95%	n/Ph+	n	n	12%	0.50%	0.00%	54,000	9000	5000
14	85%	Ph+	Ph+/n	n/Ph+	50%	32.50%	15.50%	19,000	10,000	10,100
15	90%	Ph+	Ph+/n	n	59%	37.60%	4.10%	26,000	12,000	9000
16	87%	Ph+/n	n/Ph+	n	33.30%	5.60%	0.00%	27,000	8500	8000
17	99%	n	n	n	15.10%	5.40%	0.10%	32,000	11,000	9000
18	80%	n/Ph+	n	n	11.12%	0.06%	0.00%	31,800	9000	9000
19	85%	n/Ph+	n	n	40.47%	8.60%	0.00%	14,400	11,000	10,000
20	99%	n	n/Ph+	n	13.50%	6.16%	0.00%	40,000	7000	9600
21	85%	Ph+	Ph+/n	Ph+/n	45%	31%	15%	30,000	12,000	9000
22	99%	n/Ph+	Ph+	Ph+/n	15%	29%	24.20%	15,000	8000	12,300
23	90%	Ph+/n	n/Ph+	n	25.80%	12.66%	0.07%	21,000	9800	9000
24	95%	Ph+/n	Ph+/n	n/Ph+	36%	17.12%	3.50%	72,000	11,000	10,000
25	90%	n	n/Ph+	n	50%	32%	0.30%	32,000	9000	10,200
26	37%	n/Ph+	n	n	10%	2.50%	1.50%	30,000	12,000	10,000
27	80%	n	n	n	9.70%	3.60%	0.73%	43,000	25,000	6000
28	55%	n	n	n	6.26%	1.45%	0.26%	60,000	7000	8000
29	65%	n/Ph+	n	n	1.80%	1.50%	0.90%	45,000	8000	5000
30	30%	n	n	n	10%	5.30%	90.00%	32,000	9000	7000
31	91%	n	n	n	14.20%	4.40%	80.00%	35,000	19,000	21,000
32	29%	Ph+/n	n/Ph+	n	8.00%	40.00%	0.00%	23,000	12,000	9000
33	95%	n/Ph+	n	n	1.60%	0.20%	0.40%	110,000	10,000	8000
34	91%	n	n	n	2.61%	0.05%	0.00%	16,000	5000	8000
35	67%	n	n	n	7.21%	6.40%	0.00%	20,000	9000	9500

**TABLE 5:** Monitoring CML previously diagnosed patients with molecular, cytogenetic and total leucocyte count results and FISH values calculated from the predicted‐FISH.

NO.	CCE Monitoring (Karyotype)			qRT‐PCR Monitoring			WBC (10^9^\L)			Calculated FISH using Predicted‐FISH		
	> 3 months	> 6 months	> 9 months	> 3 months	> 6 months	> 9 months	> 3 months	> 6 months	> 9 months	> 3 months	> 6 months	> 9 months
1	n/Ph+	n	n	13.0%	9.0%	0.1%	12,300	8600	6400	48.6%	37.5%	0.0%
2	n	n	n	3.0%	0.3%	0.0%	6400	6600	5100	22.0%	0.0%	0.0%
3	n/Ph+	n	n	10.2%	7.5%	0.1%	13,400	8600	7600	43.8%	34.3%	0.0%
4	n/Ph+	n	n	15.6%	4.5%	0.5%	14,300	5600	6400	54.9%	24.8%	0.0%
5	n	n	n	3.3%	0.3%	0.0%	7700	5600	6500	23.9%	0.0%	0.0%
6	n	n	n	2.9%	0.0%	0.0%	8900	6500	4600	24.1%	0.0%	0.0%
7	n/Ph+	n	n	21.0%	10.0%	0.4%	17,900	6300	7700	66.4%	37.9%	0.0%
8	n/Ph+	n	n	14.0%	9.2%	0.2%	20,320	11,700	7400	56.1%	40.4%	0.0%
9	n	n	n	9.1%	5.3%	0.0%	10,600	8200	4600	39.4%	29.0%	0.0%
10	n	n	n	6.7%	0.1%	0.0%	8600	6700	5800	32.4%	0.0%	0.0%
11	n	n	n	4.5%	0.2%	0.0%	7900	6400	8600	26.8%	0.0%	0.0%
12	n	n	n	2.5%	0.0%	0.1%	6100	7500	4700	20.5%	0.0%	0.0%
13	n	n	n	4.2%	0.1%	0.0%	6800	6300	7800	25.2%	0.0%	0.0%
14	n/Ph+	n	n	3.5%	0.0%	2.2%	8600	6900	6600	25.2%	0.0%	20.3%
15	n	n	n	0.0%	0.0%	0.0%	7500	7800	8400	0.0%	0.0%	0.0%
16	n	n	n	0.0%	0.1%	0.0%	5500	6500	4700	0.0%	15.1%	0.0%
17	n/Ph+	n/Ph+	n	12.6%	5.3%	0.1%	15,400	7900	6800	50.1%	28.7%	0.0%
18	Ph+/n	n	n	12.0%	7.3%	0.0%	30,500	10,700	8300	60.0%	35.6%	0.0%
19	n/Ph+	n/Ph+	n	14.7%	4.5%	0.8%	16,400	7500	8700	54.7%	26.5%	0.0%
20	n/Ph+	n/Ph+	n	26.4%	17.5%	0.0%	26,900	17,500	7900	78.4%	60.3%	0.0%
21	n/Ph+	n	n	11.0%	3.2%	0.3%	13,200	6500	5800	45.4%	22.6%	0.0%
22	n	n	n	13.0%	7.3%	2.0%	15,400	10,100	7500	50.8%	35.1%	20.6%
23	Ph+/n	n/Ph+	n	25.5%	12.0%	2.4%	40,300	18,600	11,500	83.2%	51.3%	25.4%
24	n	Ph+/n	n	5.0%	35.5%	10.2%	10,800	84,230	11,200	30.6%	100%	42.2%
25	Ph+/n	n/ph+	n	35.0%	16.6%	4.3%	32,400	18,500	6900	89.3%	59.4%	25.5%
26	n/Ph+	n/Ph+	n	13.3%	11.8%	7.0%	15,200	11,400	6500	51.2%	45.6%	31.4%
27	Ph+	Ph+/n	n	60.0%	28.1%	12.4%	96,400	43,300	11,700	100.%	86.7%	47.0%
28	n	n	n	0.5%	0.0%	0.0%	11,600	7700	7300	0.0%	0.0%	0.0%
29	n/Ph+	n	n	15.5%	10.2%	3.2%	14,400	11,500	9600	54.8%	42.4%	25.4%
30	Ph+	Ph+/n	n/Ph+	40.5%	26.6%	10.2%	72,400	28,800	13,900	98.1%	79.4%	44.2%
31	n	n	n/Ph+	5.3%	0.0%	11.7%	7500	5400	14,500	28.4%	0.0%	47.7%
32	Ph+	Ph+/n	n/Ph+	62.0%	28.0%	12.0%	77,400	32,600	13,600	85.9%	82.6%	47.6%
33	n/Ph+	n	n/Ph+	13.2%	6.4%	11.3%	16,300	7800	13,600	51.7%	31.1%	46.2%
34	Ph+	n/Ph+	n	40.0%	32.9%	3.3%	96,500	43,000	9600	100%	90.3%	25.7%
35	n/Ph+	n	n	12.3%	5.4%	0.0%	12,300	8700	5500	47.2%	29.7%	0.0%
36	n	n	n	7.7%	2.0%	0.0%	7600	7800	5400	33.9%	20.9%	0.0%
37	n/Ph+	n	n	4.0%	0.0%	0.0%	19,800	8600	4600	36.6%	0.0%	0.0%
38	n	n	n	2.4%	0.1%	0.0%	6600	6300	6900	20.6%	0.0%	0.0%
39	n/Ph+	n	n	4.1%	0.0%	0.1%	32,700	7300	4600	48.6%	0.0%	0.0%
40	n/Ph+	n	n	33.5%	12.5%	2.2%	19,500	12,400	5700	84.6%	47.7%	19.5%
41	n/Ph+	n	n	0.1%	0.0%	0.0%	12,500	7800	7400	0.0%	0.0%	0.0%
42	n	n	n	0.0%	0.0%	0.0%	10,300	7900	5700	0.0%	0.0%	0.0%
43	n/Ph+	n	n	12.0%	4.0%	0.0%	14,700	9500	7900	48.4%	27.2%	0.0%
44	n/Ph+	n	n/Ph+	18.0%	6.4%	0.1%	20,700	10,500	9500	63.1%	33.5%	0.0%
45	n	n	n	5.0%	0.0%	0.0%	5300	6900	9300	25.7%	0.0%	0.0%
46	n	n	n	12.0%	4.0%	0.1%	11,600	8600	10,400	46.1%	26.4%	0.0%
47	n/Ph+	n	n	17.0%	6.4%	0.5%	14,900	8900	8500	57.9%	32.0%	0.0%
48	n	n	n	3.0%	0.0%	0.0%	9200	6400	5400	24.6%	0.0%	0.0%
49	n	n	n	2.8%	0.0%	0.0%	4300	4200	5300	19.5%	0.0%	0.0%
50	n/Ph+	n	n	19.3%	10.3%	4.0%	16,400	11,400	7300	62.8%	42.5%	25.2%

**FIGURE 3:**
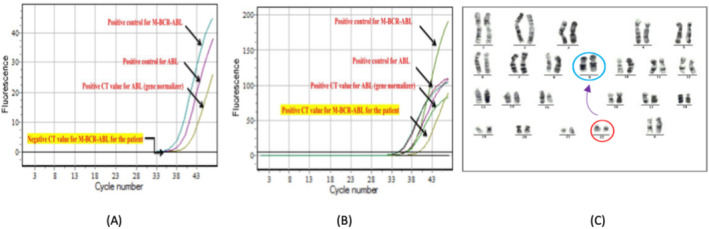
The qRT‐PCR results of M‐BCR‐ABL and M‐ABL in CML Patient Samples for (A) negative result; (B) positive result; (C) reveal abnormal karyotype image represent the standard translocations 46 XX, t (9;22) (q34; q11) which produce (BCR‐ABL1) gene.

Figure 3 shows qRT‐PCR results of M‐BCR‐ABL and M‐ABL in CML patient samples for (A) negative result from patient number (10) after 3 months of treatment; (B) positive result from patient number (1) after 3 months of treatment; and (C) abnormal karyotype image representing the standard translocations 46 XX, t (9; 22) (q34; q11) which produce (BCR‐ABL1) gene from patient number 3 at first detection.

**FIGURE 4:**
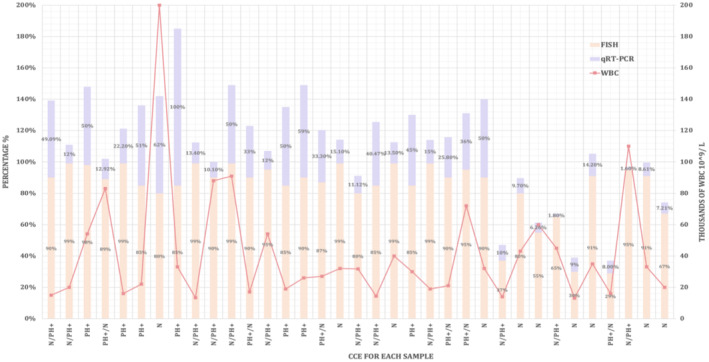
Detection of CML Newly Diagnosed Patients with Molecular, Cytogenetic and Total Leucocyte Count Results before Treatment.

Figure 4 indicates the use of multiple diagnostic methods in order to confirm the presence of CML in individuals before exposure to treatment using multiple methods such as CCE, qRT‐PCR, and WBC.

**FIGURE 5:**
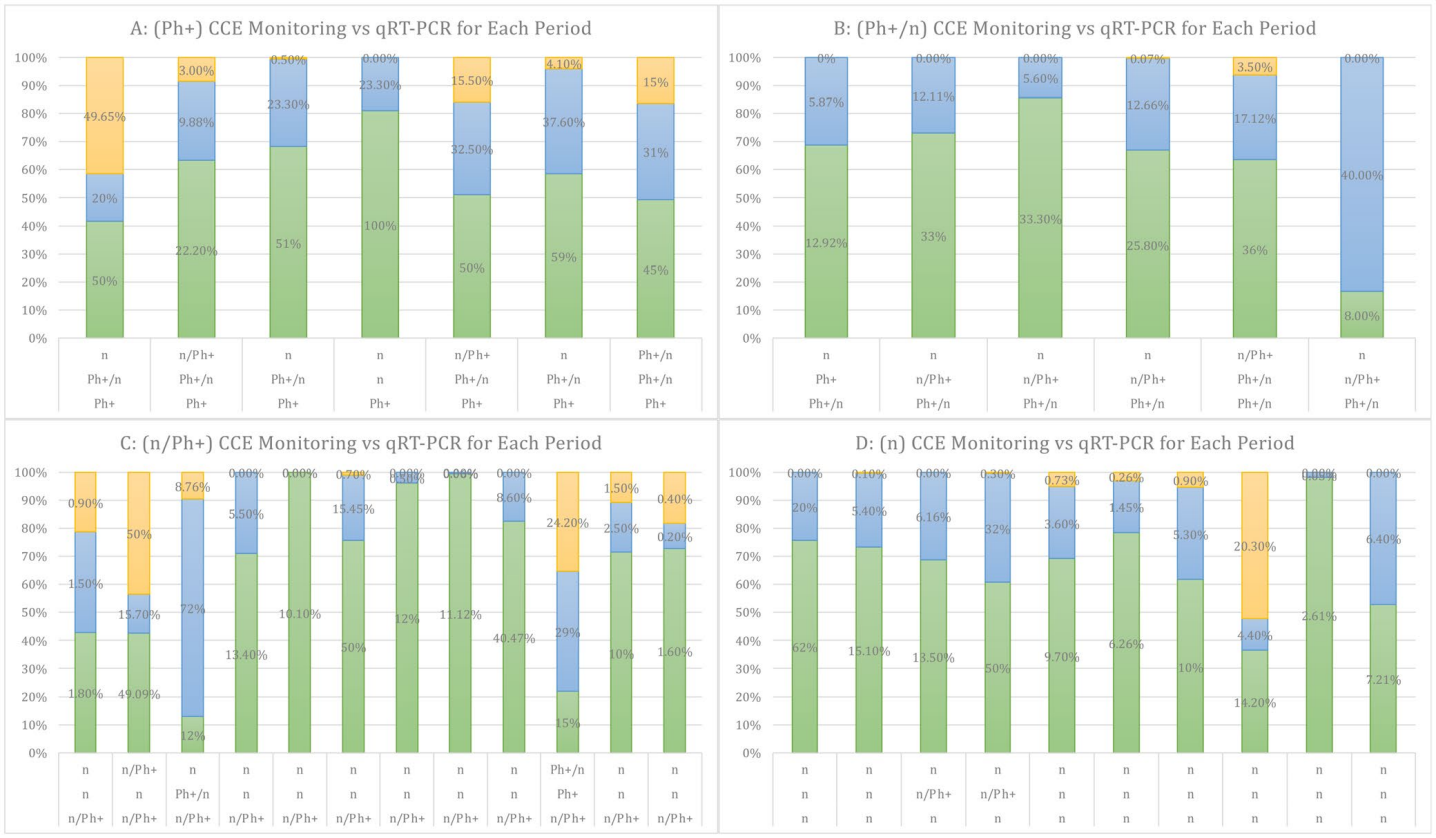
Represent Detection and Monitoring of Newly Diagnosed Patients of CML by CCE and QRT‐PCR.

Figure 5 shows the detection and monitoring of newly diagnosed CML patients using methods (CCE and qRT‐PCR).

**FIGURE 6:**
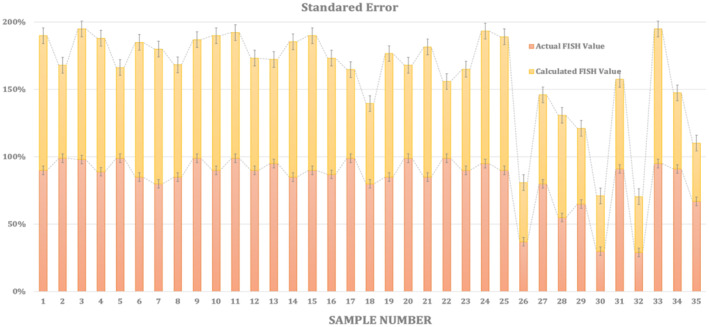
Standard Error for Actual FISH Value versus Predicted FISH for 35 patients.

Figure 6 shows the standard error comparison between the Actual FISH and the designed Predicted‐FISH.

**FIGURE 7:**
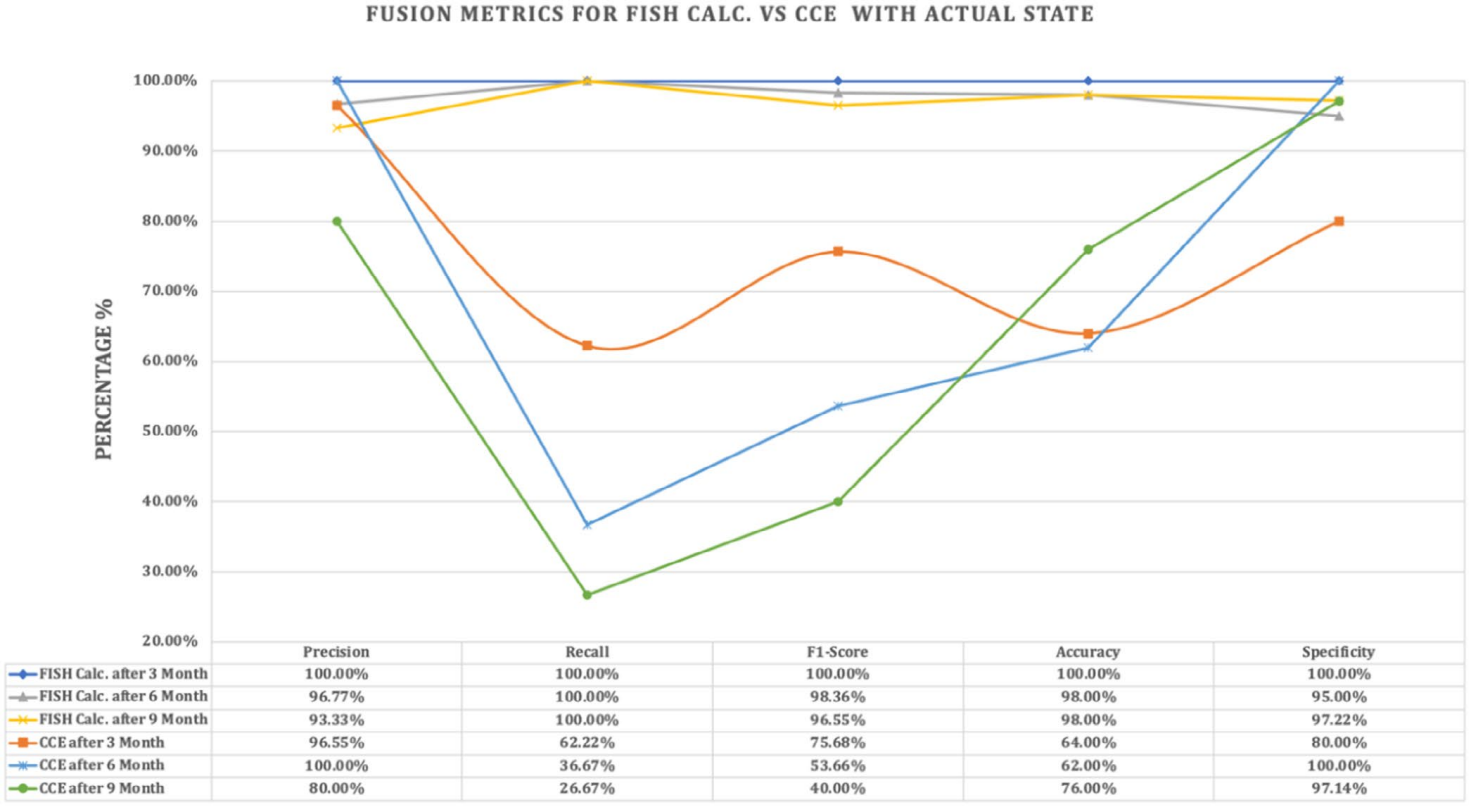
Fusion Metrix for The Predicted‐FISH versus CCE for 50 Patients for three Periods.

Figure 7 performs the fusion matrix (precision, recall, F1‐score, accuracy and specificity) for the predicted FISH versus CCE for three periods.

**FIGURE 8:**
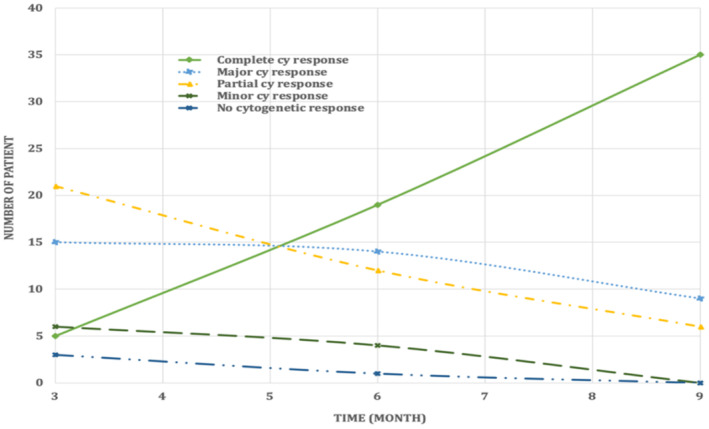
Cytogenetic Response using The Predicted‐FISH for 50 Patients over Monitoring Periods.

Figure 8 represents the cytogenetic response using The Predicted‐FISH for monitoring periods.

**FIGURE 9:**
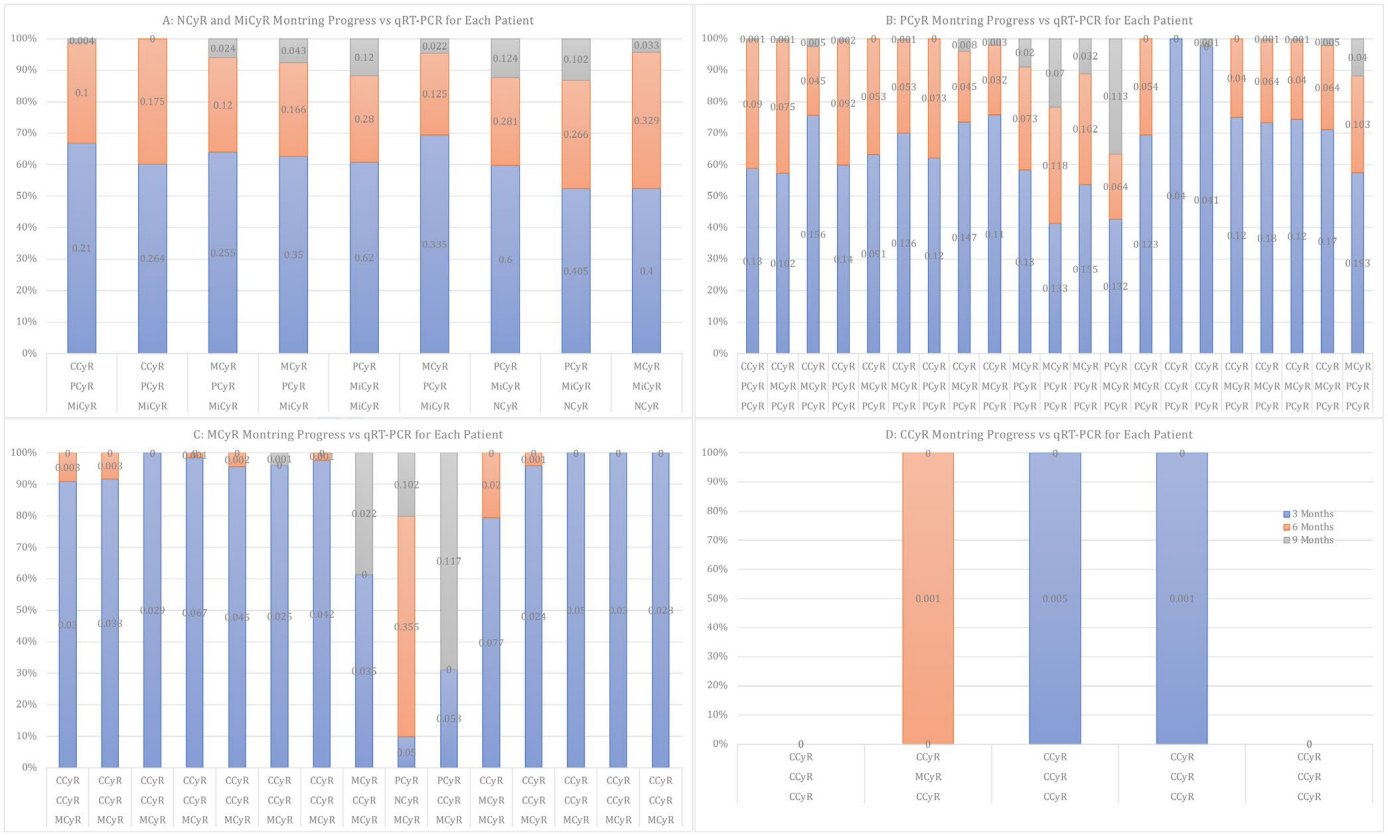
Represent Detection and Monitoring of 50 Patients of CML by The Predicted‐FISH and qRT‐PCR for three Periods.

Finally, Figure 9 represents the detection and monitoring of 50 patients of CML by the Predicted‐FISH and qRT‐PCR for three periods.

## 6 Discussion

The present study holds significant scientific importance in the research of CML by focusing on optimizing methodologies for patient monitoring and treatment evaluation. Through the use of progressive techniques such as CCE, qRT‐PCR and WBC, the study aims to enhance the accuracy and efficiency of disease detection and progression monitoring in CML patients. These methods are crucial for assessing genetic abnormalities, quantifying disease burden at the molecular level, and evaluating hematologic parameters, which collectively contribute to informed clinical decision‐making, this is supported by Hughes and Ross [20]; Baccarani et al. [21] and, Hochhaus et al. [22].

Furthermore, the study endeavours to develop a novel predictive model for interpreting fluorescence in situ hybridization (FISH) results, a technique used to detect specific chromosomal abnormalities associated with CML. By refining predictive models, the research intends to provide clinicians with enhanced tools for prognostication and treatment planning tailored to individual patient profiles. The Predicted‐FISH is directly affected by both qRT‐PCR and WBC, unlike the other variables, which are supported by Manuel et al. [23]; Mikhail et al. [24].

The study cohort included 60 males (54.55%) and 50 females (45.45%), showing an approximate male‐to‐female ratio of 1.2:1. The mean age of the patients was (37.83 ± SD) years. This is similar to the mean age reported (39.5 ± SD) in Baghdad by Yasmeen et al. [25], but lower compared to Kirkuk, where the mean age of patients was reported as (44 ± SD) Mohammed et al. [26]. Furthermore, with time, the validity of CCE will decrease at which the cytogenetic analysis appears normal in patients who are still suffering from CML. Here, qRT‐PCR is an accurate diagnostic tool, supported by Pieńkowska et al. [27].

The majority of samples exhibit varying degrees of abnormalities associated with CML, as indicated by FISH, CCE and qRT‐PCR beside WBC results as in Figure 4 above. These abnormalities primarily include the presence of Ph, observed in varying percentages across different samples. For instance, sample 3 shows a high Ph + CCE (98% FISH positivity) and (50%) BCR‐ABL1 level, suggesting a predominant abnormal CCE with significant disease involvement. In addition, Sample 8 demonstrates a significant presence of Ph + CCE (85% FISH positivity), indicating a substantial proportion of cells carrying Ph. This is confirmed by the presence of (100%) BCR‐ABL1 level in qRT‐PCR, reflecting a high disease burden. Contrastingly, Sample 7 exhibits (n) in CCE monitoring but the FISH (80%) refers to the presence of Ph + and it's confirmed by both a high BCR‐ABL1 level (62%) in qRT‐PCR and high WBC.

*qRT‐PCR monitoring* initial percentages of positive results (e.g., 62% as in Figure 5‐D) decrease dramatically over time (> 3 and > 6 months), indicating a reduction in BCR‐ABL1 levels in which this decrease correlates with improved molecular response to treatment. In *the early stage*, initial high percentages across all CCE groups and high positivity in qRT‐PCR indicate a significant disease burden at diagnosis. In the *mid‐stage*, decreasing percentages in CCE groups suggest a cytogenetic response to treatment which is supported by Zhi‐Dong et al. [28]. In addition, a significant decrease in qRT‐PCR positivity indicates effective suppression of BCR‐ABL1 levels, aligning with treatment goals. In the *late stage*, further reductions in all CCE groups and lower percentages across all categories indicate potential disease control or remission, with minimal residual disease.

From the above figure, it is clear that there are four cases of patients, three of whom showed a loss of therapeutic response, which requires intensive follow‐up with the possibility of changing the treatment or increasing the dose. They are (1, 3, 30 and 31) with a percentage of (50%, 49.65%, 90.00% and 20.3%) of qRT‐PCR at a period of 6 months, while the fourth showed resistance to the disease, which is the patient (22) with a percentage (29%) of qRT‐PCR within 3 months, which requires changing treatment and intensive follow‐up as well.

CCE analysis revealed a great number of abnormal cells and a small number of normal cells during the mitotic phase according to the mitotic index in the field, and the results were in agreement with those observed by Luciano et al. [29]. Abnormal cells showing an irregular pattern are commonly used to indicate a sample positive for genomic rearrangements [30].

Regular use of FISH can provide clinicians with accurate and timely information on disease status which is supported by Haidary et al. [31], facilitating optimal management strategies and improving patient outcomes. Despite the error percentage between the actual FISH and the FISH model standard errors as in Figure 6 above, the Predicted‐FISH is still considered reliable and can be used for such calculations. This means that the Predicted‐FISH can provide a reasonable estimate of the standard error, even if it's not exacted which is supported by the model of [32] for detecting CML in Zebrafish or Mice. In this case, the Predicted‐FISH is likely being used to predict the treatment response of patients with different CCE statuses, as we discussed earlier.

Figure 7 above shows the Predicted‐FISH consistently has high precision across all time intervals (> 93%), indicating a low rate of false positives in identifying CML‐positive cases. While, CCE initially demonstrates comparable precision (> 96%) but shows a decline over time (> 9 months), suggesting increasing difficulty in accurately identifying positives without false positives. Also, the Predicted‐FISH maintains perfect recall (100%) throughout, indicating it consistently identifies all true positive cases of CML. Whereas, CCE initially has lower recall (> 62%) and decreases significantly over time (> 9 months), indicating it misses a substantial proportion of true positive cases as the disease progresses. Moreover, the Predicted‐FISH achieves high F1 scores (> 96%) across all time intervals, reflecting its balanced performance in precision and recall. While, CCE shows a decline in the F1‐score over time (> 9 months), indicating reduced effectiveness in disease detection compared to the Predicted‐FISH, these results are supported by Baccarani et al. [9]; Gamal and Samira [33].

Last but not least, the Predicted‐FISH maintains high accuracy (> 98%) consistently, indicating its overall correctness in identifying both positive and negative cases of CML. CCE shows a slight decline in accuracy over time (> 6 months), although it improves (> 9 months), indicating varying effectiveness in correctly identifying cases as the disease progresses. Finally, the Predicted‐FISH and CCE, methods demonstrate high specificity (> 80%), suggesting their ability to correctly identify true negative cases of CML without false positives. The Predicted‐FISH emerges as the more reliable method for CML diagnosis, consistently demonstrating higher precision, recall, F1‐score, and accuracy compared to CCE. While both methods maintain high specificity, the decline in recall and F1‐score for CCE over time (> 6 and > 9 months) underscores its limitations in detecting CML progression effectively. The superior performance of the Predicted‐FISH in identifying CML cases supports its use for timely treatment decisions and monitoring disease progression.

The number of patients achieving CCyR, as in Figure 8 above, increases steadily from 3 months (5 patients) to 9 months (35 patients), indicating an improving trend in eliminating Ph. MCyR and PCyR show fluctuations but generally decrease over time, reflecting ongoing efforts to reduce cytogenetic abnormalities. MiCyR decreases over time, with no patients achieving MiCyR by 9 months, indicating the difficulty in achieving even minor reductions in cytogenetic abnormalities. NCyR decreases over time, reflecting overall treatment efficacy in most patients, these results are supported by Michael et al. [34].

In general, from Figure 9 above the 50 patients show progress by treatment as a cytogenetic response is enhanced except for three patients. They are (14 and 31) with a percentage of (22.0% and 11.7%) of qRT‐PCR at a period of 9 months showing loss MMR in the 9th month, while the third sample (24) showed resistance to the disease, with a percentage. (35.5%) of qRT‐PCR within 6 months, which requires changing treatment and intensive follow‐up as well, these results are supported by Shah et al. [35], Mahon et al. [36].

## 7 Conclusions

It is clear, that this study emerges that the FISH technique is the most accurate in detecting the disease. While CCE showed fairly good results in monitoring and diagnosing cured patients, it showed poor accuracy in diagnosing patients' responses to treatment over time. QRT‐PCR is considered excellent in monitoring patients with CML and indicating the level of presence and quantity of the BCR‐ABL1 gene through the detection of MMR and DMR. The Predicted‐FISH concluded very accurate results in detecting the disease at a rate of more than (98%), with better sensitivity in small percentages, as well as very good accuracy (82.7%) in detecting the CML disease severity. This helps in diagnosing the extent of the patient's response to treatment and the decision to change the dose or the treatment for the patient better.

## Conflicts of Interest

The authors declare no conflicts of interest.

## Data Availability

The datasets used and/or analyzed during the current study are available from the corresponding author upon reasonable request.
